# Patients presenting with lower urinary tract symptoms who most deserve to be investigated for primary bladder neck obstruction

**DOI:** 10.1038/s41598-021-83672-5

**Published:** 2021-02-18

**Authors:** Nicolò Schifano, Paolo Capogrosso, Rayan Matloob, Luca Boeri, Luigi Candela, Giuseppe Fallara, Antonio Costa, Edoardo Pozzi, Federico Belladelli, Walter Cazzaniga, Costantino Abbate, Francesco Montorsi, Andrea Salonia

**Affiliations:** 1 Università Vita-Salute San Raffaele, Milan, Italy; 2 Division of Experimental Oncology/Unit of Urology, URI Urological Research Institute, IRCCS Ospedale San Raffaele, Via Olgettina 60, 20132 Milan, Italy; 3 Department of Urology, IRCCS Foundation Ca’ Granda Ospedale Maggiore Policlinico, University of Milan, Milan, Italy

**Keywords:** Urology, Bladder, Urological manifestations

## Abstract

We aimed to investigate clinical features potentially useful in primary bladder neck obstruction (PBNO) diagnosis in men presenting with lower urinary tract symptoms (LUTS). Data from 1229 men presenting for LUTS as their primary complaint at a single centre were retrospectively analysed. All patients underwent a comprehensive medical and physical assessment, and completed the International Prostate Symptoms Score. All patients were investigated with uroflowmetry, and trans-rectal ultrasound imaging to define prostate volume. Urodynamic evaluation was performed when the diagnosis of benign prostatic enlargement was not confirmed and the patient presented a significant chance of detrusor overactivity or underactivity. As per our internal protocol, patients < 60 years old with bothersome LUTS and > 60 years with a prostate volume (PV) < 40 mL were also investigated with urethrocystoscopy to rule out urethral stricture. Logistic regression analysis tested clinical predictors of possible PBNO. Of 1229 patients, 136 (11%) featured a clinical profile which was consistent with PBNO. Overall, these patients were younger (*p* < 0.0001), had lower BMI (*p* < 0.0001), less comorbidities (*p* = 0.004) and lower PSA values (*p* < 0.0001), but worse IPSS scores (*p* = 0.01) and lower PV values (*p* < 0.0001) compared to patients with other-aetiology LUTS. At multivariable analysis, younger age (OR 0.90; *p* = 0.003) and higher IPSS scores (OR 1.12; *p* = 0.01) were more likely to be associated with this subset of patients, after accounting for other clinical variables. One out of ten young/middle-aged men presenting for LUTS may be affected from PBNO. Younger patients with more severe LUTS systematically deserve an extensive assessment to rule out PBNO, thus including urethrocystoscopy and urodynamics with voiding-cysto-urethrogram.

## Introduction

Male lower urinary tract symptoms (LUTS) have traditionally been related to bladder outlet obstruction (BOO), which is often associated with a benign prostate enlargement (BPE), resulting from the histologic condition of benign prostatic hyperplasia (BPH)^1^. However, LUTS could be also the results of conditions unrelated to the prostate itself^2^. In this context, primary bladder neck obstruction (PBNO), dysfunctional voiding-pseudodyssynergia, detrusor under-activity or over-activity, neurogenic bladder dysfunction, urethral strictures and urinary infections have all been described as causes of LUTS, especially in young men^1^. PBNO, also called congenital bladder neck sclerosis or Marion’s disease, is characterized by failure of the bladder neck to open adequately during voiding, resulting in obstructed urinary flow in the absence of anatomical obstruction associated with benign prostate enlargement (BPE)^3,4^. Its estimated prevalence is 47–54% among young male patients with LUTS^5,6^. The aetiology is unknown and a range of theories have been proposed^4^. Crowe et al.^7^ found an increase in the density of neuropeptide Y-immunoreactive nerves in bladder neck specimens of affected patients, thus suggesting a neurological role for the failure of the bladder neck to funnel properly. Others have noted a difference in bladder neck morphology, whereby an aberrant orientation of the bladder neck muscle fibers is thought to result in a pathologic narrowing of the bladder neck during a detrusor contraction^8^. PBNO may present with voiding or storage symptoms, or a combination of both^9^. Another feature that can be associated with PBNO is pain, which was reported to be more prevalent for male vs. female patients^9^. For these reasons, PBNO is frequently misdiagnosed with chronic prostatitis, neurogenic or psychogenic bladder voiding dysfunction^9^, eventually leading to a delay in the delivery of appropriate treatments. Videourodynamic assessment is the most accurate diagnostic tool for the investigation of voiding dysfunction issues, especially in young men. A videourodynamic diagnosis of PBNO is confirmed when a number of features are co-existent, including: narrowing only at the vesical neck on a fluoroscopic voiding cystourethrogram; relatively high detrusor pressure during voiding; obstructive flow pattern; and relaxed external sphincter electromyography. Even if conservative options are similar (e.g., *α*-blockers) for those patients with anatomical obstructions (e.g., BPE) and for PBNO-patients, the surgical management differs between the former and the latter subsets of BOO-patients. Whilst *α*-blocker treatment is highly useful for controlling the LUTS in BPE-patients, these medications have shown lower levels of efficacy in men with PBNO. Furthermore, a number of studies identified the existence of long-term compliance issues with these medications (e.g., with only 24–30% of patients continuing the treatment after 1 year)^4^. Indeed, PBNO surgical treatment is mainly based on bladder neck incision, whilst the surgical management of the other BOO-patients should be addressed according to the site of anatomical obstruction. To the very best of our understanding, no studies to date specifically looked at the risk of developing long-term complications such as renal function impairment due to detrusor underactivity. In this context, an early diagnosis of PBNO is thought to contribute in controlling these possible detrimental outcomes^4^. As such, defining clinical predictors of PBNO would be useful to identify patients who deserve to be investigated and managed for this condition. Hence, we aimed at identifying a range of clinical predictors suggesting a PBNO condition in men seeking medical help for LUTS at a single academic outpatient centre.

## Materials and methods

We identified 1323 patients presenting for voiding or mixed LUTS as their primary complaint at a single-centre outpatient clinic and assessed by the same senior physician between 2009 and 2019. This retrospective chart review study involving human participants was in accordance with the ethical standards of the institutional and national research committee and with the 1964 Helsinki Declaration and its later amendments or comparable ethical standards. The study was approved by the local ethical committee (IRCCS OSR Prot. 2014 – Pazienti Ambulatoriali). All individual partecipants included in the study signed an informed consent form and agreed to share their own anonymous information for future studies**.**

All patients underwent a comprehensive diagnostic work-up. Maximal effort has been employed to reduce missing data in the design of the study, and complete-case analysis method was adopted to address this issue. Clinical variables collected included age, health-significant comorbidities, as scored by the Charlson Comorbidity Index (CCI)^10^, calculated body mass index (BMI) and previous/concomitant medical therapies. A comprehensive physical examination was performed to exclude external-meatal stenosis, phimosis and other abnormalities of the external genitalia. A digital-rectal examination (DRE) was performed in all cases.

All patients were invited to complete the International Prostate Symptoms Score (IPSS) questionnaire. Moreover, patients were also requested to complete the International Index of Erectile Function-erectile function domain (IIEF-EF)^11^ and the Beck’s Inventory for Depression questionnaire (BDI)^12^.

Urinary and semen culture were performed to exclude urinary infection; total prostate-specific antigen (PSA) was assessed in men aged > 45 years. Moreover, all patients were investigated with uroflowmetry, and trans-rectal ultrasound (TRUS) imaging to define prostate volume (PV). Urodynamics, including filling cystometry and pressure-flow study was also performed when the diagnosis of BPE was not confirmed and the patient presented with a significant chance of detrusor overactivity or underactivity^13^. Young age (e.g. < 60 years), low urinary flow (e.g. Qmax < 10 ml/s), low voided volume (e.g. < 150 ml) and high postvoidal residual volume (e.g. > 300 ml) were considered suggestive of detrusor underactivity^13^. The role of cystourethroscopy in the assessment of PBNO-patients is important, although this examination is not sufficient by itself in confirming a definitive PBNO-diagnosis. In fact, endoscopic assessment is helpful in ruling out alternative diagnoses such as urethral strictures, posterior urethral valves, inflammatory lesions, or foreign bodies^14^. As per our internal protocol, patients younger than 60 years old with bothersome LUTS were usually further investigated with urethrocystoscopy to rule out urethral stricture. Likewise, patients older than 60 years with a PV < 40 mL were investigated with urethrocystoscopy since smaller prostate may be less likely the cause of BOO^15^. According to this protocol a total of 72% of the included patients underwent endoscopic assessment. Endoscopic features considered suggestive of PBNO were as follows: internal urethral sphincter hypercontraction and complete lack of elasticity in the absence of urethral strictures posterior urethral valves, inflammatory lesions, or foreign bodies^14^. The diagnosis of possible PBNO also required values of peak flow rate (PFR) consistent with outflow obstruction (e.g., PFR < 10 ml/s, provided that a sufficient 150-mL threshold of voided volume was achieved)^13^.

Overall 83 (6.3%) and 11 (0.8%) patients were excluded from the analysis because of previous prostate surgery or history of urethral stricture, respectively, thus resulting in a cohort of 1,229 (92.9%) patients eligible for statistical analyses.

### Statistical analyses

Patients’ characteristics are presented as medians (inter quartile ranges (IQR)). Descriptive statistics with Kruskall-Wallis and Fisher’s exact test were used to analyse significant differences in clinical parameters between patients with and without a clinical profile suggestive of PBNO. Clinically significant variables were used to build a logistic regression model testing clinical predictors of PBNO.

Given that a urethrocystoscopy was not performed in all patients older than 60 years, with 28% of cases who did not underwent an endoscopic assessment, we may have misdiagnosed some cases of PBNO in the older population. As such, we performed a sensitivity analysis testing our predictive model only in patients who were screened with urethrocystoscopy (N = 879 (72%)). Statistical analyses were conducted using Stata 13.0 (StataCorp, College Station, TX, USA), with a two-sided significance level set at *p* < 0.05.

## Results

Table 1 shows baseline characteristics of the entire cohort. Median (IQR) age at first assessment was 59 (43, 68). Of all, 18% of patients depicted at least one comorbid condition at baseline. Out of 1229 patients, 136 (11%) had a clinical profile consistent with PBNO, whilst a BOO caused by BPH was observed in 1042 (84.7%) cases. Other causes of LUTS were over-active bladder syndrome in 19 (1.6%) of cases and chronic prostatitis in 32 (3.6%) of cases (Table 1). As for LUTS severity, total IPPS scores were suggestive for mild, moderate and severe LUTS in 25%, 49%, and 26% of patients, respectively (Table 1).

**Table 1:** Descriptive characteristics of the entire cohort (N. = 1229).

Age yrs [Median (IQR)]	59 (43, 68)
BMI [Median (IQR)]	24.8 (22.7, 27.0)
**CCI [N. (%)]**	
0	1008 (82)
≥ 1	221 (18)
PSA ng/mL [Median(IQR)]	1.9 (0.8, 4.3)
TRUS-PV mL [Median(IQR)]	57 (32, 91)
PFR ml/s [Median (IQR)]	13 (8.8, 18.6)
**Diagnosis of PBNO [N. (%)]**	
No	1093 (89)
Yes	136 (11)
**Other causes of LUTS [N. (%)]**	
BPH	1042 (84.7)
OAB	19 (1.6)
Chronic prostatitis	32 (3.6)
IPSS total [Median (IQR)]	13 (7, 20)
**LUTS severity (according to IPSS) [N. (%)]**	
Mild (< 8)	307 (25)
Moderate (8–19)	602 (49)
Severe (> = 20)	320 (26)
IIEF-EF [Median (IQR)]	24 (9, 29)
BDI score [Median (IQR)]	5 (2, 9)
**Depressive symptoms (BDI ≥ 11) [N. (%)]**	
No	1008 (82)
Yes	221 (18)

Keys: BMI = body mass index; CCI = Charlson comorbidity index; PSA = prostate-specific antigen; TRUS = trans-rectal ultrasound; PV = prostate volume; PFR = peak flow rate; PBNO = primary bladder neck obstruction; LUTS = lower urinary tract symptoms; BPH = benign prostatic hyperplasia; OAB = overactive bladder; IPSS = International Prostate Symptoms Score; IIEF-EF: International Index of Erectile Function-erectile function domain; BDI = Beck’s Inventory for Depression.

Table 2 details clinical differences between patients with a clinical profile suggestive of PBNO, and those patients where a PBNO diagnosis was excluded. Overall, possible PBNO patients were younger (42 vs. 61 yrs; *p* < 0.0001), had lower BMI (23.4 vs. 25; *p* < 0.0001), lower CCI scores (CCI ≥ 1: 10% vs. 19%; *p* = 0.004), and lower PSA (0.8 vs. 2.5 ng/mL; *p* < 0.0001), but worse IPSS scores (16 vs. 12; *p* = 0.01) and lower PV values (26 vs. 61 mL; *p* < 0.0001) compared to patients with LUTS for other reasons (Table 2). Moreover, we also observed a small but significant difference in the IIEF-EF score, which was higher for possible PBNO patients (25 vs. 24; *p* = 0.02). No differences were observed in terms of depressive symptoms and PFR (Table 2).

**Table 2:** Differences in clinical characteristics between patients with and without diagnosis of PBNO.

	Other causes of LUTS	PBNO	*p* value
[N. (%)]	1093 (89)	136 (11)	
Age yrs [Median(IQR)]	61 (47, 69)	42 (35, 48)	< 0.0001
BMI [Median (IQR)]	25.0 (22.9, 27.2)	23.4 (22.0, 24.8)	< 0.0001
**CCI [N. (%)]**			
0	885 (81%)	123 (90%)	0.004
≥ 1	208 (19%)	13 (10%)	
PSA ng/mL [Median (IQR)]	2.5 (1.1, 5.1)	0.8 (0.5, 1.3)	< 0.0001
TRUS-PV mL [Median (IQR)]	61 (39, 90)	26 (20, 41)	< 0.0001
PFR ml/s [Median (IQR)]	12.5 (8.4, 18.4)	13.0 (9.3, 16.0)	0.8
IPSS total [Median (IQR)]	12 (7, 20)	16 (11, 21)	0.01
IPSS Storage	6.0 (3.0, 9.0)	7.0 (3.5, 9.0)	0.3
IPSS Voiding	7.0 (3.0, 12.0)	9.0 (6.0, 12.5)	0.01
IIEF-EF [Median (IQR)]	24 (8, 28)	25 (15, 29)	0.02
BDI [Median (IQR)]	5.0 (2.0, 9.0)	3.0 (1.5, 7.5)	0.2
**Depressive symptoms (BDI ≥ 11) [n. (%)]**			
No	996 (81)	120 (88)	0.4
Yes	233 (19)	16 (12)	

Keys: PBNO = primary bladder neck obstruction; LUTS = lower urinary tract symptoms; BMI = body mass index; CCI = Charlson comorbidity index; PSA = prostate-specific antigen; TRUS = trans-rectal ultrasound; PV = prostate volume; PFR = peak flow rate; IPSS = International Prostate Symptoms Score; IIEF-EF: International Index of Erectile Function-erectile function domain; BDI = Beck’s Inventory for Depression.

At the multivariable logistic regression analysis adjusting for all significantly different clinical parameters (Table 3), younger age (OR 0.90; 95% CI 0.84–0.96; *p* = 0.003) and a greater LUTS severity according to IPSS scores (OR 1.12; 95% CI 1.02–1.22; *p* = 0.01) were more likely to be associated with a clinical profile suggestive of PBNO.

**Table 3:** Logistic regression analysis testing predictors of PBNO.

	OR	95% CI	*p* value
Age	0.90	0.84, 0.96	0.003
BMI	0.91	0.73, 1.12	0.4
CCI (0 vs- > = 1)	1.07	0.11, 10.80	1
PSA	0.63	0.33, 1.19	0.2
PV	0.99	0.95, 1.03	0.6
PFR	0.95	0.87, 1.04	0.3
Total IPSS	1.12	1.02, 1.22	0.01

Keys: PBNO = primary bladder neck obstruction; BMI = body mass index; CCI = Charlson comorbidity index; PSA = prostate-specific antigen; PV = prostate volume; PFR = peak flow rate; IPSS = International Prostate Symptoms Score.

**Figure 1:**
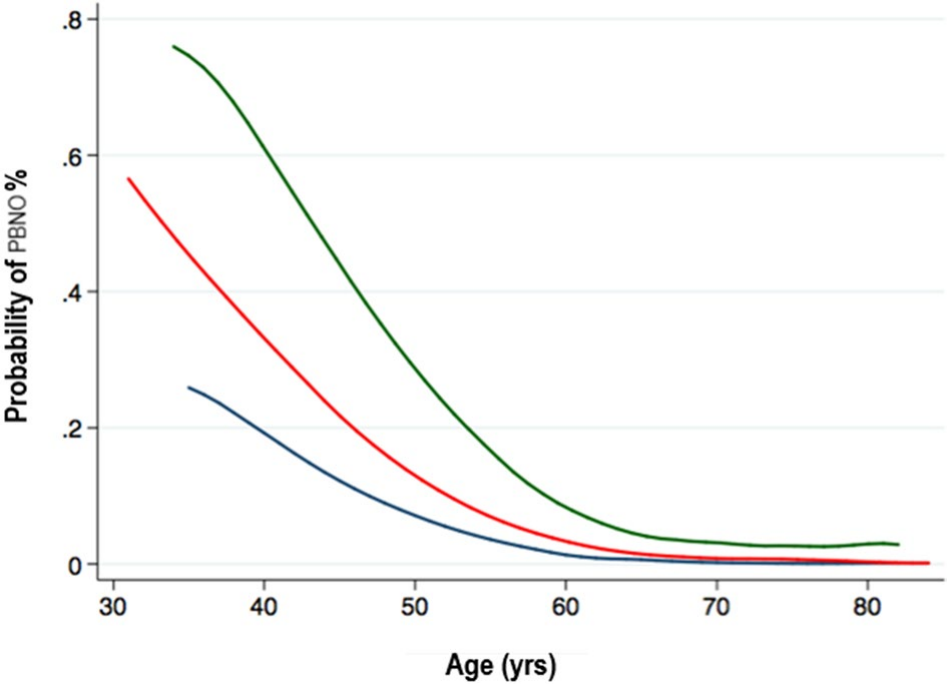
Probability of PBNO according to age. The green line represents patients with severe LUTS; the red line represents patients with moderate LUTS, the blue line represents patients with mild LUTS. Keys: PBNO = primary bladder neck obstruction; LUTS = lower urinary tract symptoms.

According to this model, the adjusted probability of suffering from PBNO for a 40-yr man with mild LUTS was 26% (95% CI 1–51), compared to 47% (95% CI 9–84) for a same-age man presenting with severe LUTS. As expected, the differences in the probability of PBNO according to different levels in LUTS severity seems to decrease for older ages which are more likely to suffered from BOO due to BPH (Fig. 1).

At sensitivity analysis, younger age and worse LUTS were confirmed as significant predictors of PBNO when testing our model only in patients who underwent an urethrocystoscopy (N = 879 (72%)) (Table 4).

**Table 4:** Logistic regression analysis testing predictors of PBNO among patients with urethrocystoscopy (N = 879 (72%)).

	OR	95% CI	*p* value
Age	0.90	0.83, 0.97	0.005
BMI	0.92	0.74, 1.14	0.4
CCI (0 vs. ≥ 1)	1.49	0.13, 16.82	0.7
PSA	0.68	0.35, 1.30	0.2
PV	0.98	0.94, 1.03	0.4
PFR	0.94	0.85, 1.04	0.2
Total IPSS	1.11	1.01, 1.22	0.03

Keys: PBNO = primary bladder neck obstruction; BMI = body mass index; CCI = Charlson comorbidity index; PSA = prostate-specific antigen; PV = prostate volume; PFR = peak flow rate; IPSS = International Prostate Symptoms Score.

## Discussion

We investigated the prevalence and clinical predictors of PBNO in a contemporary real-life same-ethnicity cohort of patients complaining of LUTS. Overall, our data showed that 11% of them had diagnostic criteria suggestive for PBNO. Of clinical interest, we found that younger patients with more severe symptoms were more likely to be associated with a clinical profile suggestive for PBNO, after accounting for other clinical factors.

To the best of our knowledge, this study provides novel findings about the prevalence of PBNO in a large cohort of men presenting for LUTS in the real-life clinical practice. So far, previous epidemiological surveys have investigated PBNO in specific very selected populations. Kaplan et al.^5^, for instance, identified a group of 137 LUTS patients. All 137 men had previously been misdiagnosed with chronic prostatitis and had been consistently treated without success; mean age of these subjects was 37 years, and 74 (54%) were diagnosed with PBNO. Nitti et al.^6^ evaluated 85 men presenting with LUTS and who agreed to undergo a complete videourodynamic evaluation. Of all, patients older than 45 years were excluded to minimize any contribution of a possible BPH. The mean age of these subjects was 37 years, and the most common diagnosis was PBNO, which was found in 40 (47%) cases. Yang et al.^16^ selected 84 men younger than 55 years presenting with both chronic voiding dysfunction and obstructive uroflowmetry patterns. The diagnosis of PBNO was made by the identification of a relaxed external sphincter electromyography during voiding, in the absence of both any distal urethral obstruction and narrowing only at the bladder neck on voiding cystourethrography. Of these, 28 (33.3%) fulfilled the criteria of PBNO. Karami et al.^17^ assessed 456 men younger than 40 years with chronic LUTS; they diagnosed PBNO in 21% of the patients, based on a diagnostic protocol similar to the one that was here proposed, which included urodynamic assessment (e.g., without the video-component), cystoscopy and TRUS.

Considering the specific inclusion criteria of the above mentioned studies, their result may be hardly translated into clinical practice in order to guide the diagnostic work-up of men complaining of LUTS in the real-life setting. In contrast, we investigated the prevalence of PBNO in a larger population of patients with LUTS, which could be potentially consequent to several conditions including BPH, chronic prostatitis, bladder dysfunction or PBNO. Of all, almost one out of ten patients demonstrated diagnostic features suggestive of PBNO.

We also analyzed specific clinical features which could guide physicians to investigate patients for PBNO. Our data suggest that in young patients suffering from severe LUTS, the diagnosis of PBNO should be pursued since they would harbor a significantly higher risk of suffering from LUTS because of this condition rather than BPH or urethral strictures. Conversely, current findings show that the probability of PBNO significantly drops after 60 years of age. In line with these finding it has been previously suggested that as men age it becomes clinically difficult to distinguish PBNO from BPH and it is possible that many men with PBNO are misdiagnosed with the latter^4^. Even in our series, we did not perform urethrocystoscopy in all men over the age of 60; indeed, as for routine practice in our department, we perform an endoscopic evaluation in older men only in the case of voiding symptoms and low PFR despite a small PV, with the specific aim to rule out other causes of BOO, such as urethral strictures and PBNO.

Clinical strengths of current observations are several folds. First, this is the first study assessing the prevalence of PBNO in a broad and not-selective cohort of patients complaining of LUTS in the everyday clinical setting. Second, we are aware that a comprehensive diagnostic assessment including urethrocystoscopy and videourodynamic investigation^4^ may not be offered to most men with LUTS, if not even to all, according to current clinical guidelines^1^. Hence, our findings suggest to perform a more in depth assessment, thus including urethrocystoscopy and videourodynamic investigation, in young men with more severe LUTS who more likely appear to suffer from BOO due to PBNO.

The current study presents with a range of limitations. First, it lacks of systematic videourodynamic assessments to confirm the diagnosis of PBNO in every man; hence, though not highly probable, those patients who received a diagnosis of PBNO could have suffered instead from a different functional impairment of the bladder neck, such as bladder neck dyssinergia or remaining conditions^14^. We acknowledge that current European Association of Urology (EAU) guidelines^1^ suggest to avoid to offer non-invasive tests such as uroflowmetry as an alternative to pressure-flow studies for diagnosing BOO. However, it is of utmost importance considering this study from an historical perspective. Uroflowmetry diagnostic performance is limited due to the inability to discriminate BOO and detrusor hypocontractility. However, based on the EAU guidelines suggestions over the years, a BOO diagnosis was considered likely for PFR < 10 ml/s, thus no urodynamic study was considered clinically useful when BOO was presumable^13^. It is a well-known issue that the indication to urodynamic investigation for patients seeking medical help for LUTS has been the subject of a lengthy debate owing to the invasive nature of urodynamic testing^13^. Clinical features such as younger (e.g., < 50 years) or older (e.g., > 50 years) age, and predominantly voiding bothersome LUTS, were considered by EAU guidelines panels^13^ useful to evaluate who could benefit the most from urodynamics. However, there were no uniform consensus neither high levels of evidence on whether urodynamic evaluation was compulsory in patients suffering from LUTS^13^. Therefore, as per our internal protocol, this investigation was offered at our institution in those situations in which the diagnosis of BPE was not confirmed and there was a significant suspicion of detrusor overactivity or underactivity.

Finally, as a single center study our results needs external validation in larger multicenter studies.

## Conclusions

Primary bladder neck obstruction should be always considered throughout the diagnostic work-up of men presenting with LUTS. In this context, younger patients with more severe LUTS may deserve a more comprehensive assessment to rule out PBNO, thus including urethrocystoscopy and urodynamic assessment with voiding cysto-urethrogram.

## Data availability

The data that support the findings of this study are available from the corresponding author, AS, upon reasonable request.
